# Canine testicular tumors: two types of seminomas can be differentiated by immunohistochemistry

**DOI:** 10.1186/s12917-014-0169-8

**Published:** 2014-08-06

**Authors:** Marko Hohšteter, Branka Artuković, Krešimir Severin, Andrea Gudan Kurilj, Ana Beck, Ivan-Conrado Šoštarić-Zuckermann, Željko Grabarević

**Affiliations:** 1 Department of Veterinary Pathology, Veterinary Faculty, University of Zagreb, Heinzelova 55, Zagreb 10 000, Croatia; 2 Department of Forensic and Judicial Veterinary Medicine, Veterinary Faculty, University of Zagreb, Heinzelova 55, Zagreb 10 000, Croatia

**Keywords:** Dog, Testis, Tumors, Seminoma, Immunohistochemistry, Incidence, IGCNU

## Abstract

**Background:**

Testicular tumors are the most common genital neoplasms in male dogs, with Leydig cell tumors (LCT), seminomas (SEM), and Sertoli cell tumors (SCT) the most common forms. Human SEM are classified as classical (CSEM) or spermatocytic (SSEM). Intratubular germ cell neoplasia of undifferentiated origin (IGCNU) is another form of human testicular tumor. The aim of this study was to verify that CSEM/SSEM classification is valid in dogs and confirm the existence of canine IGCNU.

**Results:**

Testicular tumors were found in 46% of dogs at necropsy and accounted for 7% of tumors biopsied. The median age of dogs with tumors at necropsy was 10.16 years; median age at positive biopsy was 10.24 years. The most common tumors, in decreasing order, were LCT, mixed tumors, SEM and SCT at necropsy, and SEM, SCT, mixed tumors, LCT, peripheral nerve sheath tumor, and teratoma in the biopsy group. IGCNU was found in 3% of testicles at necropsy and in 3% of biopsy samples. Two dogs had testicular tumor metastasis. Expression of c-KIT was most common in SEM and seminomatous components of mixed tumors. PLAP was mostly expressed in IGCNU, SEM, teratoma, and some mixed tumors. Cytokeratin was mainly expressed in SCT. CD30 expression was low in both groups.

**Conclusions:**

The high tumor incidence at necropsy can be attributed to older age. Tumor incidence in biopsy samples, dog age, and histological classification were consistent with previous studies. The higher incidence of SEM and SCT in the biopsy group probably resulted from the obvious clinical expression of these tumor types. The low incidence of metastasis confirmed the predominance of benign tumors. Low CD30 expression confirmed the low incidence of testicular embryonal carcinoma. Cytokeratin helps differentiate stromal tumors, especially SCT, from germ cell tumors. Histology and c-KIT and PLAP expression indicate that IGCNU exists in dogs. Expression of c-KIT and PLAP confirmed that CSEM and SSEM classification is valid in dogs.

## Background

Testicular tumors are the most common neoplasms of the genital system in male dogs and are the third most common type of canine tumor after skin and fibrous tissue tumors [1]. Testicular tumors represent more than 90% of all canine male genital tumors and dogs have the highest incidence of all animal species [2].

Investigations of the incidence of testicular tumors in dogs at necropsy have shown somewhat discordant results. One dated study found an incidence of 16% [3], while a more recent paper reported an incidence as high as 27% [4]. According to research conducted by Gamlem et al., testicular tumors represented 7% of all biopsied tumors in dogs from 1990 to 1998 [5]. In another study, Vescalari et al. [6] found that male genital tumors represented 13% of all biopsied tumors of male dogs from 2005 to 2008. About 40% of neoplastic testicles have more than one tumor [7]. Testicular tumors are often classified as mixed tumors, although they actually result from two different tumor types occurring in the same testis [8].

Primary testicular tumors are histologically classified into germ cell tumors, sex cord-stromal (gonadostromal) tumors, and mixed germ cell-sex cord stromal tumors [9]. Within these groups are the three most common canine testicular tumors, which have relatively similar incidence varying by study. Sertoli cell tumors (SCT) and Leydig cell tumors (LCT) are sex cord-stromal tumors and seminomas (SEM) are germ cell tumors [1],[4],[10].

Many immunohistochemical markers are used for differentiation of human testicular tumors and although some of them have been studied in canine testicular tumors, information about them is still insufficient. c-KIT is used in human patients for differentiation of c-KIT positive CSEM from c-KIT negative SSEM [11],[12]. c-KIT is also expressed in human IGCNU contrary to nonseminomatous germ cell tumors and stromal tumors which do not express c-KIT [11]-[13]. In dogs some studies have shown that a certain percentage of SEM express c-KIT [10],[13],[14] which is in disagreement with other reports that describe the absence or very rare c-KIT expression in canine SEM [15],[16]. PLAP is widely used marker that is expressed in human IGCNU and very often in human CSEM [11],[12]. Investigations by Grieco et al. [17] and Yu et al. [10] confirmed the expression of PLAP in some canine SEM, but there are no data on PLAP expression in canine IGCNU. Cytokeratin AE1/AE3 is used in human diagnostics as a marker for the differentiation of cytokeratine negative testicular germ cell tumors from cytokeratine positive embryonal carcinoma, yolk sac tumors and other carcinomas. Cytokeratin is also expressed in SCT and LCT [11],[18]. In veterinary medicine, there are only few reports of cytokeratin expression in testicular tumors. In all of them SEM showed no immunoreactivity to cytokeratin which is expressed mainly in SCT and mixed SCT, and rarely in LCT [14],[19]. According to results of Banco et al. [19] cytokeratin is not expressed in normal Sertoli cells, so this marker can be useful for differentiation of SCT, not only from other testicle tumors but also from neoplastically unaltered Sertoli cells. CD30 shows high expression in human simple and mixed testicular embryonal carcinoma and is used for differentiation of this tumors from other germ cell tumors [11],[12]. Like in humans, investigation by Yu et al. [10] confirmed the expression of CD30 in canine embryonal carcinomas.

Doubts have been raised in recent studies about the classification of SEM in dogs. Some studies have shown that SEM in dogs, as in humans, can be classified into two types: classical (CSEM) and spermatocytic (SSEM) [10],[17],[20]. In contrast, Bush et al. [15] and Thorvaldsen [16], found that canine SEM are predominantly spermatocytic, suggesting that there are no (or extremely rare) cases of canine CSEM.

In humans, CSEM is the predominant SEM type, with a high incidence among young men. CSEM originates from transformed gonocytes (prespermatogonia and spermatogonia), while SSEM are neoplasms of older men and are derived from more differentiated germ cells, mostly spermatocytes [15],[21]-[24]. It is probable that this different origin of SSEM determines its predominantly benign behavior, in contrast to CSEM, which is malignant with a high metastatic potential [20],[25]. Canine SEM is mostly benign; however, it does metastasize in a small number of cases [26].

It is also not clear whether intratubular germ cell neoplasia of undifferentiated origin (IGCNU) or *carcinoma in situ* is found in canine testicles. These tumors are very common as precursor lesions of CSEM in men, and recently some authors have suggested that identical tumors can be observed in canine testicles [17],[27],[28]. In humans, IGCNU is similar to CSEM, and according to some reports canine CSEM is derived from gonocytes (prespermatogonia) and spermatogonia. These cells express the germ cell markers c-KIT and PLAP. SSEM, which is derived from more differentiated cells, namely spermatocytes, does not or only focally expresses c-KIT and PLAP [10],[11],[13],[17],[20],[27],[29]-[31].

The aim of this study was to determine usefulness of immunohistochemical markers (c-KIT, PLAP, cytokeratin, CD30) in differentiation of canine testicular neoplasia. Further objectives included verification that differentiation between CSEM and SSEM is valid in dogs and confirmation of the existence of canine IGCNU.

## Methods

### Tissue specimens and clinical data

This study was approved by the Ethics committee of Veterinary faculty, University of Zagreb. Archived biopsy samples collected from April 2007 through January 2011 from 52 dogs (59 testicles) were analyzed at the Department of Veterinary Pathology, University of Zagreb. Most biopsy specimens were from dogs surgically treated at the Clinics of the Veterinary Faculty, while a smaller number were from private practices throughout Croatia. The dogs’ ages at the time of the surgery were in the range of 2–15 years (mean, 10.24 years; one was of unknown age).

Samples from 170 macroscopically normal and abnormal testicles were also collected from 85 dogs routinely necropsied at the Department of Veterinary Pathology, University of Zagreb from October 2009 through December 2011. The ages of necropsied dogs with testicular tumors were in the range of 1–18 years (mean, 10.16 years; one was of unknown age). Dogs in both groups were of various pure and mixed breeds.

### Histological examination

Samples were fixed in 10% neutral buffered formalin. Some of the biopsy samples were delivered already formalin-fixed. Samples were embedded in paraffin wax and 5-μm sections were stained with hematoxylin-eosin (HE) for histopathological examination. Stained sections were classified according to the diagnostic criteria proposed by the World Health Organization (WHO) [32]. All samples were also analyzed for the presence of IGCNU. Periodic acid-Schiff staining (PAS) was used for better visualization of PAS-positive vacuoles in testicles with diagnosed IGCNU.

### Immunohistochemistry

Eighty necropsy samples and 50 biopsy samples were selected for immunohistochemical analysis. All selected samples were representative specimens of testicles with tumors previously diagnosed by examination of HE-stained samples. Immunohistochemical analyses were also conducted on one sample from all histologically normal testicles. Immunohistochemistry was not conducted on highly autolytic samples.

Immunohistochemical analyses were conducted using the avidin-biotin complex method. For immunohistochemical analyses, monoclonal mouse anti-human PLAP, anti-human CD30, anti-human cytokeratin AE1/AE3, and polyclonal rabbit anti-human c-KIT antibodies were used. All antibodies were produced by DAKO (Glostrup, Denmark). Assays were performed on 4-μm sections of paraffin-embedded tissue samples. The sections were dewaxed in xylene and rehydrated through a series of graded alcohol solutions. Antigen retrieval was carried out for PLAP and CD30 by microwave treatment (650 W) with ethylenediaminetetraacetic acid buffer, pH 9 (DakoCytomation) for 4 × 5 min. Antigen retrieval for c-KIT was carried out by microwave treatment (650 W) with TRS (Dako Target Retrieval Solution, S1700) for 20 min, and for cytokeratin AE11/AE3 with proteinase (Dako Proteinase K, S3004) at room temperature for 5 min. Sections were incubated with primary antibodies as follows: anti-human PLAP (Dako, M7191) diluted 1:25 for 30 min at room temperature; anti-human c-KIT (Dako; A4502) diluted 1:400 for 30 min at room temperature; anti-human CD30 (Dako, M0751) diluted 1:20 for 30 min at room temperature, and anti-human cytokeratin (Dako, clone AE1/AE3, M3515) diluted 1:50 for 30 min at room temperature. Initial incubation was followed by incubation for 30 min with a ready-to-use secondary antibody (Dako REAL™ EnVision™ /HRP, Rabbit/Mouse) and with the substrate Dako REAL™ Diaminobenzidine + Chromogen for a further 10 min. Samples were rinsed with DakoCytomation Wash Buffer between steps. The sections were counterstained with hematoxylin and mounted. Sections from human placenta were used as positive controls for human PLAP and human CD30, sections from human uterus for cytokeratin AE11/AE3, and sections from human seminoma for human c-KIT. Primary antibody was replaced with phosphate-buffered saline for the negative control.

### Evaluation of immunohistochemical reactions

Immunohistochemical reactions were evaluated by light microscope at 40× and 100× magnification to evaluate the percentage of positive tumor cells (range: 0–100%). Cellular distribution (nuclear, cytoplasmic, membranous) of the stain was evaluated under 400× magnification [33].

### Statistical analysis

Statistical analysis was performed using the MedCalc® program 10.2.0.0 (MedCalc Software bvba, Mariakerke, Belgium). Basic statistical analysis of results was conducted using usual methods of descriptive statistics with assessment of arithmetic mean, minimum and maximum values, geometric mean, median, and standard deviation. Normality tests were performed by the Kolmogorov–Smirnov test. Statistically significant differences of data between analyzed groups with normal distribution were evaluated by the one-way analysis of variance (ANOVA). For groups with abnormal distribution of data, Kruskal–Wallis analysis of variance was used. Analysis of statistically significant differences between groups were performed using parametrical and nonparametrical tests of significance. Values of *p <* 0.05 were considered statistically significant.

## Results

### Clinical findings and histopathological analysis

During the sampling period testicular tumors were found in 39 (46%) of 85 necropsied dogs, and in 50 (29%) of 170 testicles. In 11 dogs (28%), tumors were found in both testicles. In biopsy samples from living patients, tumors were found in 55 (93%) of 59 testicles and in 51 (98%) of 52 dogs and comprised 7% of all biopsied canine tumors during the sampling period. Four dogs (7%) had tumors in both testicles. The mean age of necropsied dogs was 8.29 years; those with testicular tumors had a mean age of 10.16 years and those without tumors had a mean age of 6.46 years. The mean age of dogs that underwent testicular biopsy was 10.06 years; the mean age of dogs with tumors was 10.24 years, and the only dog in that group without a testicular tumor was 1 year of age. Mixed-breed dogs, Labrador retrievers, poodles, golden retrievers, Alaskan malamutes, and mastiffs were overrepresented in the necropsy group. Mixed-breed dogs, golden retrievers, German shepherds, Pekingese, Yorkshire terriers, and Labrador retrievers were overrepresented in the biopsy group. Bilateral tumors were most common in mixed-breed dogs in both groups. As many as 9% of testicular tumors from biopsy samples were diagnosed in cryptorchid testicles. There was only 1 cryptorchid testicle in the necropsy group, and it did not contain neoplasia.

**Figure 1:**
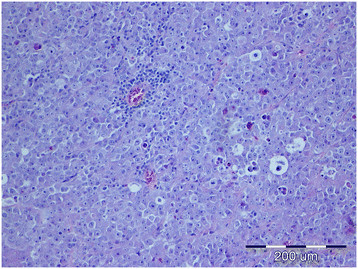
Diffuse seminoma, testicle, dog, HE, ×20.

**Figure 2:**
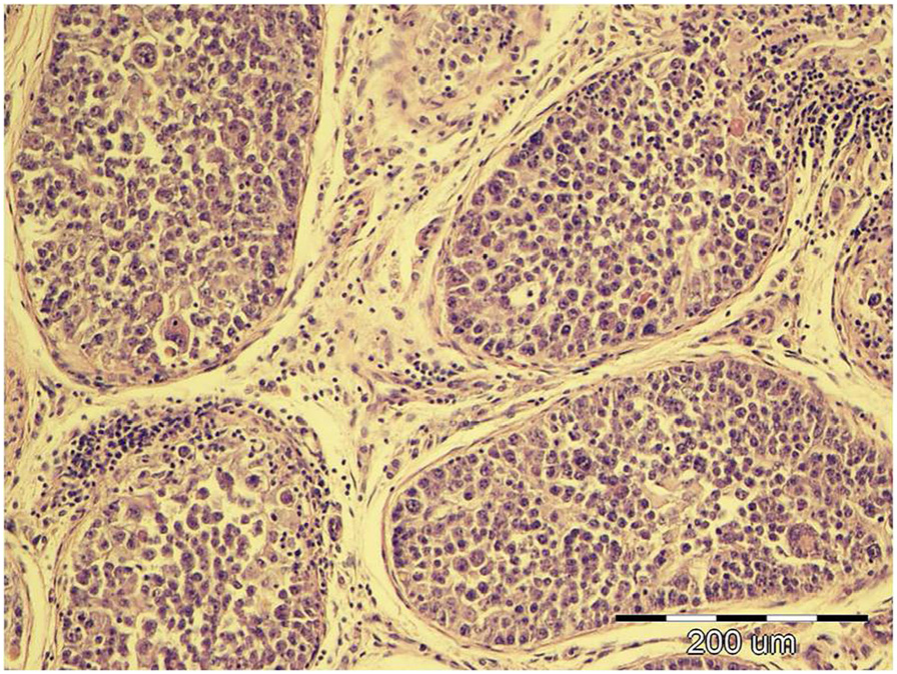
Intratubular seminoma, testicle, dog, HE, ×20.

**Figure 3:**
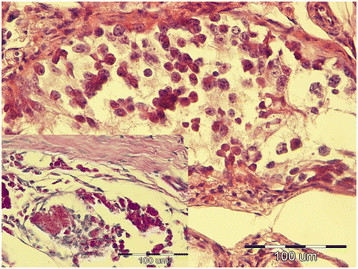
Intratubular germ cell neoplasia of undifferentiated origin (IGCNU), testicle, dog, HE, ×40; Inset: IGCNU, testicle, dog, PAS, ×40.

Tumors were classified according to the WHO classification of testicular tumors of dogs (Table 1). In the necropsy group, there were 37 (74%) simple tumors with the following incidence: 19 (38%) LCT; 11 (22%) SEM, [7 (14%) intratubular, 4 (8%) diffuse] (Figures 1 and 2); and 7 (14%) SCT. Twelve (24%) mixed tumors were found, with an incidence as follows: 5 (10%) mixed LCT/SCT, 5 (10%) mixed LCT/SEM, and 2 (4%) mixed SCT/SEM. One dog in this group had intratesticular lymphoma. Among 46 (84%) simple tumors in the biopsy group, 22 (40%) were SEM [20 (36%) diffuse, 2 (4%) intratubular], 16 (29%) SCT, 5 (9%) LCT, 2 (4%) peripheral nerve sheath tumors (PNST), and 1 (2%) teratoma. Nine mixed tumors represented 16% of all biopsied tumors with the following incidence: 4 (7%) mixed SCT/SEM, 4 (7%) mixed LCT/SEM, and 1 (2%) mixed LCT/SCT. Embryonal carcinomas were not diagnosed. Although IGCNU is not classified as neoplasia in the WHO classification of dog tumors, lesions morphologically similar to IGCNU (Figure 3) were found as sole lesions in 6 (3%) testicles of necropsied dogs and in 2 (3%) biopsied testicles. PAS-positive reactions (Figure 3) were obtained in 2 (33%) of the 6 from the necropsy group and in 1 (50%) of the 2 from the biopsy group. Testicular tumor metastases were found in only 2 dogs, both in the necropsy group: SCT in the visceral (left kidney) and parietal peritoneum, and metastasis of DIF SEM in the inguinal lymph nodes. Based on these findings, only those tumors were characterized as malignant and the rest from both groups were characterized as benign. The dog with metastatic SCT was a 15-year old mixed breed which was euthanized due to signs of testicular tumor. The dog with metastatic SEM was a 6-year old mastiff cross which was euthanized because of acute posterior paralysis. Macropathological and histopathological examination of the mastiff cross showed urine retention and dural ossification of the lumbar spinal cord with degenerative myelopathy.

**Table 1:** Incidence of histological types of testicular tumors diagnosed

**Histological type of tumor**	**Necropsy group**		**Biopsy group**	
	**Number of tumors**	**Percentage of all tumors/%**	**Number of tumors**	**%**
SIMPLE TUMORS (∑)	37	74	46	84
LCT	19	38	5	9
SCT	7	14	16	29
IT SEM	7	14	2	4
DIF SEM	4	8	20	36
PNST	0	0	2	4
Teratoma	0	0	1	2
MIXED TUMORS (∑)	12	24	9	16
LCT/SCT	5	10	1	2
LCT/ IT SEM	3	6	1	2
SCT/DIF SEM	2	4	4	7
LCT/DIF SEM	2	4	3	5
MET. T. LYMPH	1	2	0	0
**Total**	50	100	55	100

Note: ∑, sum; DIF SEM, diffuse seminoma; IGCNU, intratubular germ cell neoplasia of undifferentiated origin; IT SEM, intratubular seminoma; LCT, Leydig cell tumors; MET. T. LYMPH, metastatic tumor – lymphoma; PNST, peripheral nerve sheat tumor; SCT, Sertoli cell tumor.

### Immunohistochemical analysis

**Figure 4:**
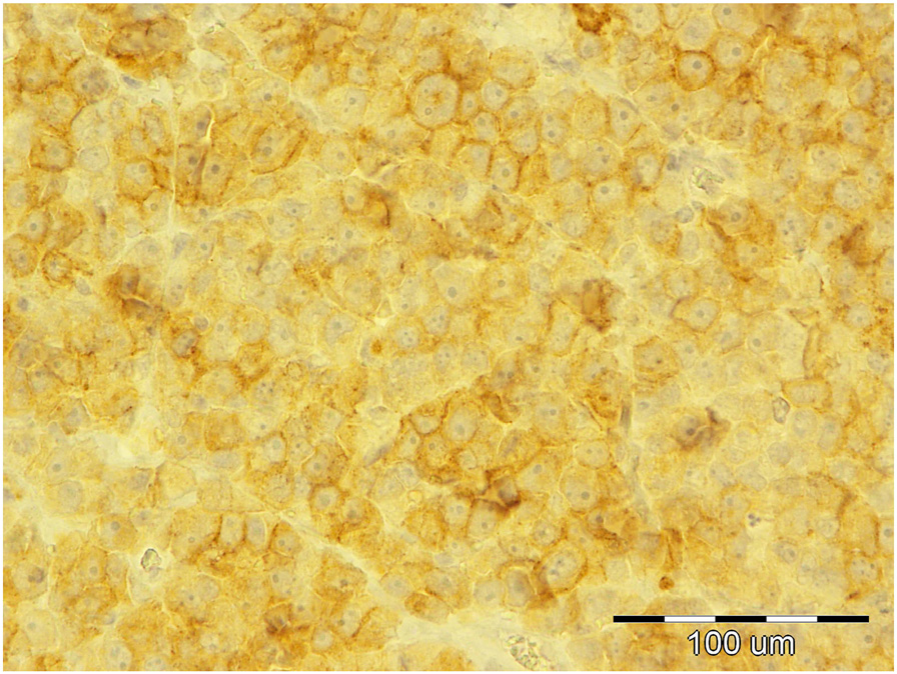
Diffuse seminoma, testicle, dog, c-KIT, ×40.

Of testicles with neoplastic changes (including IGCNU), 16% in the necropsy group and 26% in the biopsy group expressed c-KIT (Table 2). Expression of c-KIT was predominantly cytoplasmic and membranous, with moderate intensity. In both groups, SEM and the seminomatous components of mixed tumors were most often c-KIT positive. In the necropsy group, 3 (27%) of 11 SEM (Figure 4) [2 (50%) of 4 diffuse SEM; 1 (14%) of 7 intratubular SEM), 1 (16%) of 6 IGCNU, 1 (50%) of 2 mixed LCT/diffuse SEM, 1 (33%) of 3 mixed LCT/intratubular SEM, 1 (50%) of 2 mixed SCT/diffuse SEM, 1 (50%) of 2 mixed LCT/SCT, and 1 (5%) of 19 LCT] were c-KIT positive. The SEM that had metastasized was c-KIT positive. The percentage of positive cells had a range of 90% in diffuse SEM to 5–20% in intratubular SEM, IGCNU, and mixed tumors. In the biopsy group, c-KIT expression was as follows: 9 (40%) of 22 SEM [9 (45%) of 20 diffuse SEM, 0 (0%) of 2 intratubular SEM], 1 (6%) of 16 SCT, 1 (100%) of 1 teratoma, and 4 (100%) of 4 mixed LCT/SEM. The percentage of positive cells was 43% in diffuse SEM, 26% in mixed LCT/SEM, 20% in teratoma, and 5% in SCT.

**Table 2:** Expression of IHC markers by diagnosed histological type of testicular tumor

**Histological type of tumor**	**Necropsy group**								**Biopsy group**							
	**c-KIT positive tumors/%**	**% of c-KIT positive cells**	**PLAP positive tumors/%**	**% of PLAP positive cells**	**Cytokeratin positive tumors/%**	**% of Cytokeratin positive cells**	**CD 30 positive tumors/%**	**% of CD 30 positive cells**	**c-KIT positive tumors/%**	**% of c-KIT positive cells**	**PLAP positive tumors/%**	**% of PLAP positive cells**	**Cytokeratin positive tumors/%**	**% of Cytokeratin positive cells**	**CD 30 positive tumors/%**	**% of CD 30 positive cells**
SIMPLE TUMORS (∑)	11	39	23	<5	27	19	7	8	22	40	27	<6	33	43	8	23
MIXED TUMORS (∑)	33	23	25	<5	66	25	8	5	44	26	22	<7	44	46	11	30
**LCT** simple	5	5	21	<5	15	16	5	10	0	0	16	<5	20	60	6	30
with SCT	20	20	40	<5	60	23	20	5	0	0	0	0	100	50	0	0
with IT SEM	33	10	0	0	60	17	0	0	100	20	100	10	100	5	0	0
with DIF SEM	50	60	0	0	40	40	0	0	75	28	0	0	0	0	0	
**SCT** simple	0	0	28	<5	71	32	14	10	6	5	31	<5	62	51	12	27
with LCT	20	20	40	<5	60	23	20	5	0	0	0	0	100	50	0	0
with DIF SEM	50	5	50	5	100	30	0	0	0	0	25	5	50	65	25	30
**IT SEM** simple	14	5	14	5	28	5	0	0	0	0	33	10	33	5	0	0
with LCT	33	10	0	0	60	17	0	0	100	20	100	10	100	5	0	0
**DIF SEM** simple	50	90	25	5	25	5	25	5	45	43	15	11	10	12	0	0
with SCT	50	5	50	5	100	30	0	0	0	0	25	5	50	65	25	30
with LCT	50	60	0	0	40	40	0	0	75	28	0	0	0	0	0	
PNST	0	0	0	0	0	0	0	0	0	0	0	0	0	0	0	0
TERAT	0	0	0	0	0	0	0	0	0	20	0	<5	100	50	100	10
IGCNU	16		33	<5	16	5	0	0	0	0	100	5	0	0	0	0
MET. T. LYMPH	0	0	0	0	0	0	0	0	0	0	0	0	0	0	0	0
Total	16	32	23	<5	35	21	7	7	26	36	26	<6	29	44	8	25

Note: ∑, sum; DIF SEM, diffuse seminoma; IGCNU, intratubular germ cell neoplasia of undifferentiated origin; IT SEM, intratubular seminoma; LCT, Leydig cell tumors; MET. T. LYMPH, metastatic tumor – lymphoma; PLAP, placental alkaline phosphatase; PNST, peripheral nerve sheat tumor; SCT, Sertoli cell tumor; TERAT, teratoma.

**Figure 5:**
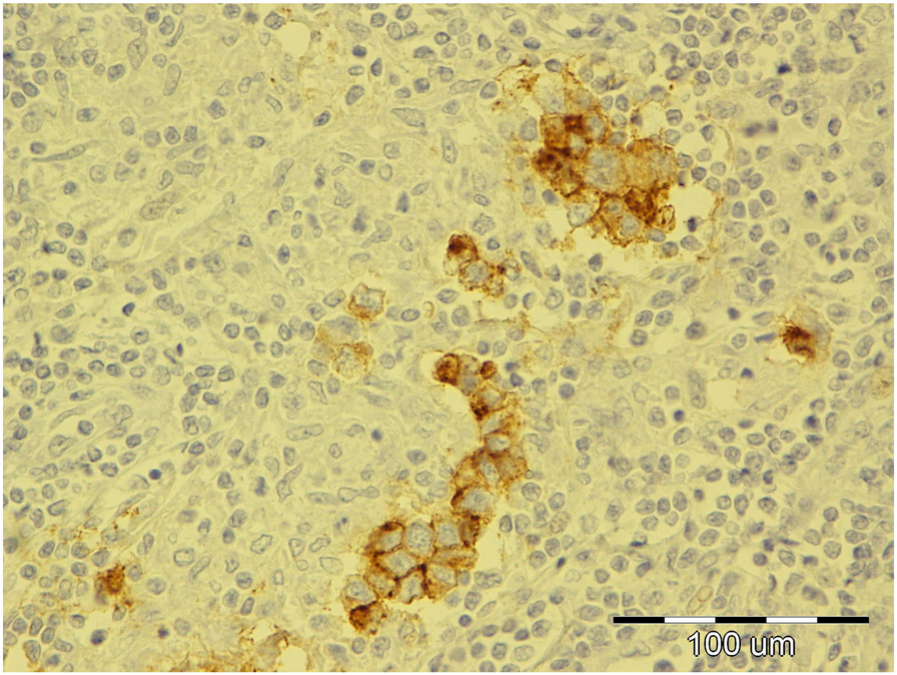
Intratubular germ cell neoplasia of undifferentiated origin, testicle, dog, PLAP, ×40.

Immunohistochemical analysis showed PLAP expression in 23% of testicular tumors (including IGCNU) from necropsied dogs and 26% of tumors (including IGCNU) from the biopsy group (Table 2). PLAP expression was predominantly cytoplasmic and membranous, with moderate intensity. The distribution of neoplastic cells with PLAP expression was mostly focally intratubular. Expression of PLAP was most common in the necropsy group IGCNU (2/6, 33%) (Figure 5), biopsied intratubular SEM (1/3, 33%), biopsied teratoma (1/2, 50%), and in some mixed tumors from both groups (25–100%). The incidence of PLAP-positive diffuse SEM was 25% in the necropsy group and 15% in the biopsy group. Both IGCNU tumors (100%) from the biopsy group showed PLAP expression.

There were 5% PLAP-positive cells in SEM and mixed SCT/diffuse SEM in the necropsy group and 5% PLAP-positive cells in biopsied IGCNU. The percentage of PLAP-positive cells in biopsied intratubular SEM and mixed LCT/intratubular SEM was 10%, and the percentage was 11% in biopsied diffuse SEM. In other tumor types, expression was lower than 5%, or absent.

Co-expression of c-KIT and PLAP was seen in 2 (10%) of 20 diffuse SEM in the biopsy group and in 1 (25%) of 4 in the necropsy group. Only 1 mixed intratubular SEM/LCT tumor from the biopsy group showed simultaneous co-expression of c-KIT and PLAP.

**Figure 6:**
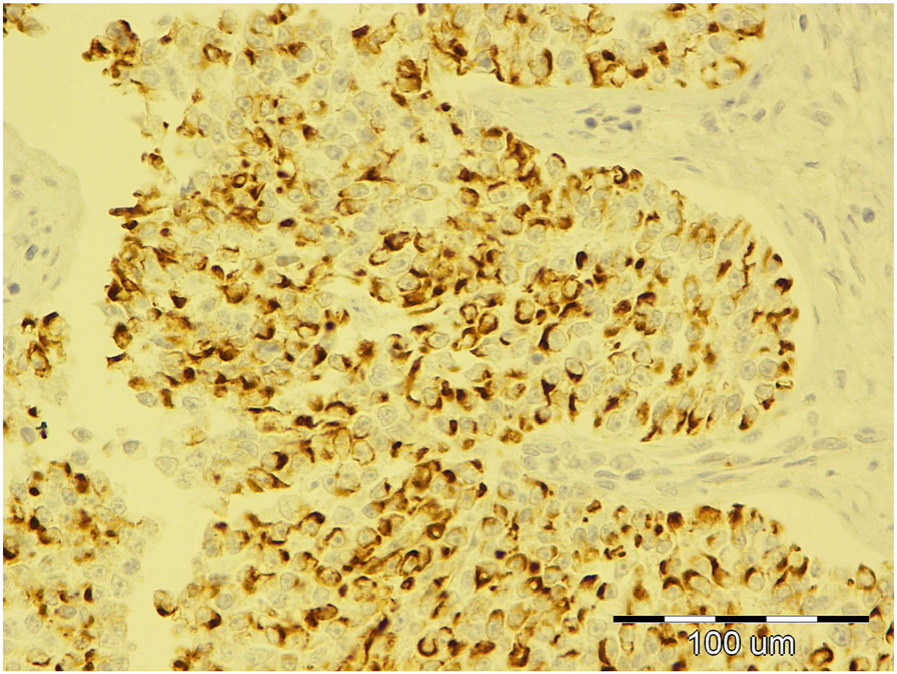
Sertoli cell tumor, testicle, dog, cytokeratin, ×40.

**Figure 7:**
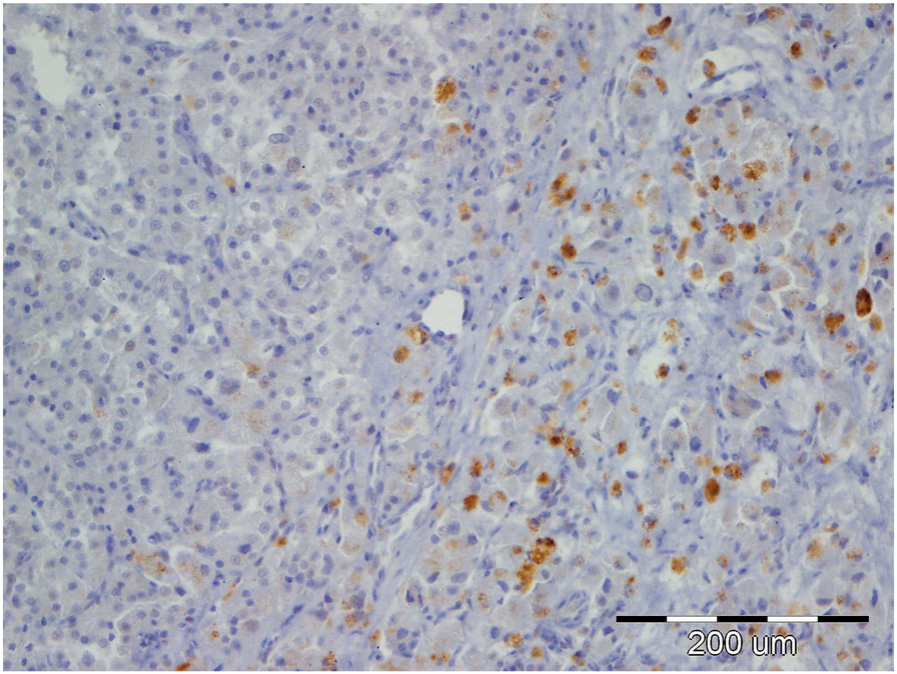
MIX SCT/LCT; cytokeratin negative Leydig cell tumor (left side of picture) and cytokeratin positive Sertoli cell tumor (right side of picture), testicle, dog, cytokeratin, ×20.

Cytokeratin was expressed in 35% of testicular tumors from necropsy samples and in 33% of biopsied testicular tumors (Table 2). Cytokeratin expression was predominantly cytoplasmic with moderate to low intensity. In both groups, positive neoplastic cells were predominantly observed in simple and mixed SCT (Figure 6). The SCT that had metastasized to the peritoneal cavity and left kidney was cytokeratin positive. In mixed SCT from both groups, cytokeratin positivity was found predominantly in the SCT components. Cytokeratin positivity was also noted in a few simple and mixed LCT. In necropsy samples, the expression of cytokeratin was as follows: 2 (100%) of 2 mixed SCT/diffuse SEM, 5 (71%) of 7 SCT, 2 (66%) of 3 mixed LCT/intratubular SEM, 3 (60%) of 5 mixed LCT/SCT, 1 (50%) of 2 mixed LCT/diffuse SEM, 2 (28%) of 7 intratubular SEM, 1 (25%) of 4 diffuse SEM, 1 (16%) of 6 IGCNU, and 3 (15%) of 19 LCT. Cytokeratin positivity was expressed in biopsy samples as follows: 1 (100%) of 1 teratoma, 1 (100%) of 1 mixed LCT/SCT (Figure 7), 1 (100%) of 1 mixed LCT/intratubular SEM, 10 (62%) of 16 SCT, 2 (50%) of 4 mixed SCT/diffuse SEM, 1 (33%) of 3 intratubular SEM, 1 (20%) of 5 LCT, and 2 (10%) of 20 diffuse SEM. Percentages of cytokeratin-positive cells were in the range of 5–12% in germ cell neoplasia from both groups. The percentages of positive cells from stromal cord tumors in the biopsy group were 65% in mixed SCT/diffuse SEM, 60% in LCT, 51% biopsied SCT, and 50% in teratomas. In the necropsy group, the percentages of cytokeratin-positive cells were as follows: 40% in mixed LCT/diffuse SEM, 32% in SCT, 30% in mixed SCT/diffuse SEM, 17% in mixed LCT/intratubular SEM, and 16% in LCT. In the necropsy group, the differences in expression of cytokeratin between SCT and intratubular SEM, SCT and LCT, and LCT and mixed LCT/intratubular SEM were significant (*p* < 0.05). In the biopsy group, the difference in expression of cytokeratin between SCT and diffuse SEM was statistically significant (*p <* 0.05).

Expression of CD30 was detected in 7% of tumors from necropsy and 8% of biopsied tumors (Table 2). Expression was cytoplasmic and membranous with low intensity. In both groups, the proportion of tumors expressing CD30 was low for nearly all tumor types, as was the percentage of positive cells.

## Discussion

Testicular tumors represented 7% of all biopsied tumors during the study time period. In our investigation, testicular tumors were found in 46% of dogs at necropsy. The high incidence of tumors in necropsied dogs cited in the literature [3],[4] is at least partially due to the relatively advanced age of dogs at the time of necropsy, because older age is a predisposing factor for testicular tumors [7],[34]. Histological tumor classification showed that the most prevalent neoplasms in dogs at necropsy were LCT, followed by SEM and SCT. The most prevalent neoplasms in the biopsy group were SEM, followed by SCT and LCT. In both groups, other simple tumors were rarely diagnosed but the incidence of mixed tumors was relatively high.

The relative incidence of tumors in the biopsy group, the age of dogs with tumors, and the results of histological classification from both groups are consistent with results from earlier reports [1],[4],[5],[35],[36].

The higher incidence of SEM and SCT in the biopsied group can be attributed to the fact that these tumor types result in more obvious clinical signs (testicular enlargement in SEM, hormonal imbalance in SCT) [4].

Even though IGCNU is not classified as a neoplastic lesion in the WHO classification of canine tumors, we did detect some morphological changes consistent with IGCNU in both groups of dogs, findings similar to those of Grieco et al. [27]. A positive PAS reaction confirmed the histological findings of IGCNU. Although the reaction was not positive in all samples, this is probably because of the small size of IGCNU, which made it difficult to prepare additional histological slides with the same changes for different staining methods.

The small number of metastatic changes found confirms that testicular tumors in dogs have predominantly benign biological behavior, although they can have malignant histological appearance [26].

Our immunohistochemical analysis showed that the investigated markers are useful for differentiation of testicular tumors in dogs. Cytokeratin AE1/AE3 showed particularly good results in differentiating stromal tumors, especially SCT, from germ cell tumors.

Low expression of CD30 in all tumors showed that embryonal carcinoma, which is mostly CD30-positive in people [37],[38], did not appear in either group and is a very rare testicular neoplasm in dogs. Focal CD30 positivity in some germ cell tumors (SEM and teratoma) is an interesting finding and should be considered for the possibility of transforming neoplastic cells into embryonal carcinoma cells, as described in human patients [39].

Our findings of positive c-KIT expression in 27% of SEM from the necropsy group (50% of diffuse SEM, 14% of intratubular SEM) and 40% of biopsied SEM (45% of diffuse SEM, 0% of intratubular SEM) are consistent with literature reports [4],[10],[14], in which germ cell tumors can express c-KIT. Higher expression of c-KIT in diffuse SEM than in intratubular SEM may be related to differences in the biological behavior of the different types. In humans c-KIT expression is highly correlated to biological behavior. CSEM are malignant type of SEM because they originate from undifferentiated c-KIT positive transformed primordial germ cells and gonocytes (prespermatogonia and spermatogonia). In contrast to CSEM, SSEM are c-KIT negative and have benign behavior, which is in accordance with their origin from differentiated germ cells, mostly spermatocytes [15],[20]-[25].

We hypothesize that in dogs the more aggressive diffuse type of tumor, like in human CSEM, expresses c-KIT more frequently because of its different cellular origin than the less invasive intratubular SEM. The higher percentage of c-KIT-positive SEM in the biopsy group can be explained by the higher incidence of diffuse SEM in that group because of its more obvious clinical symptoms (testicular enlargement) in contrast to intratubular SEM, which generally does not cause enlargement.

Expression of PLAP and to a lesser degree c-KIT in IGCNU in both groups supports the histological findings and the hypothesis that IGCNU can be found as one variety of neoplastic change in canine testicles.

The lower expression of PLAP compared with c-KIT in SEM is consistent with a study published by Yu et al. [10], in which the number of PLAP-positive SEM was much lower than c-KIT-positive tumors. The low co-expression of c-KIT and PLAP in SEM could result from the different cellular origin of seminomas, because c-KIT is expressed in both spermatogonia and prespermatogonia whereas PLAP is expressed only in prespermatogonia, while in people both cells give rise to CSEM [10],[11],[17],[20],[27],[29],[31]. Based on our results and those of Yu et al. [10], canine CSEM are probably predominantly derived from spermatogonia and to a lesser degree from prespermatogonia, given the expression of c-KIT and PLAP.

## Conclusions

Our histopathological and immunohistochemical analyses (c-KIT and PLAP expression) indicate that, just as in human classification, some canine SEM can be classified as CSEM. We conclude that c-KIT positive SEM, or at least tumors with simultaneously expressed c-KIT and PLAP can definitively be classified as CSEM. On the basis of these results, canine SEM can be divided in two groups: the less prevalent CSEM and the more prevalent SSEM.

Although canine SEM are mostly benign and rarely metastasizes [26], differentiation of canine SEM in to CSEM and SSEM on the basis of c-KIT expression may be clinically significant. Considering the more aggressive behavior of c-KIT positive CSEM in men, close clinical monitoring would be advisable for dogs with this type of tumor. However further studies are necessary to establish whether c-KIT expression is correlated with a higher metastatic rate in canine SEM.

## Abbreviations

ANOVA: Analysis of variance

CSEM: Classical seminoma

HE: Hematoxylin and eosin

IGCNU: Intratubular germ cell neoplasia of undifferentiated origin

IHC: Immunohistochemistry

LCT: Leydig cell tumors

MET. T. LYMPH: Metastatic tumor – lymphoma

PAS: Periodic acid-Schiff

PLAP: Placental alkaline phosphatase

SD: Standard deviation

SEM: Seminoma

SSEM: Spermatocytic seminoma

TERAT: Teratoma

WHO: World Health Organization

## Competing interests

The authors declare that they have no competing interests.

## Authors’ contributions

MH participated in the design of this study, performed histopathological and IHC analysis, performed necropsies, evaluated IHC, and prepared the manuscript. KS performed statistical and image analysis. BA and ŽB participated in the design of the study and histopathological analysis and necropsies. AGK and AB performed histopathological analysis and necropsies. ICŠZ performed necropsies and IHC analysis. All authors read and approved the final manuscript.
